# Evaluation of Ethnic Variations in Visceral, Subcutaneous, Intra-Pancreatic, and Intra-Hepatic Fat Depositions by Magnetic Resonance Imaging among New Zealanders

**DOI:** 10.3390/biomedicines8060174

**Published:** 2020-06-25

**Authors:** John Zhiyong Yang, Dech Dokpuang, Reza Nemati, Kevin Haokun He, Andy Baige Zheng, Maxim S. Petrov, Jun Lu

**Affiliations:** 1 School of Science, Faculty of Health and Environmental Sciences, Auckland University of Technology, Auckland 1010, New Zealand; john.yang@aut.ac.nz (J.Z.Y.); dech.dokpuang@aut.ac.nz (D.D.); 2 Division of Medical Technology, School of Allied Health Sciences, University of Phayao, Phayao 56000, Thailand; 3 Canterbury Health Laboratories, Canterbury District Health Board, Christchurch 8022, New Zealand; reza.nemati@cdhb.health.nz; 4 Saint Kentigern College, Pakuranga, Auckland 2010, New Zealand; k.he@student.saintkentigern.com; 5 Caulfield Grammar School, St Kilda East, Melbourne 3146, VIC, Australia; andyzheng123@outlook.com; 6 School of Medicine, Faculty of Medical and Health Sciences, University of Auckland, Auckland 1023, New Zealand; 7 School of Public Health and Interdisciplinary Studies, Faculty of Health & Environmental Sciences, Auckland University of Technology, Auckland 1010, New Zealand; 8 Institute of Biomedical Technology, Auckland University of Technology, Auckland 1010, New Zealand; 9 Maurice Wilkins Centre for Molecular Biodiscovery, Auckland 1010, New Zealand; 10 College of Food Engineering and Nutrition Sciences, Shaanxi Normal University, Xi’an 710119, China; 11 College of Life Sciences and Oceanography, Shenzhen University, Shenzhen 518071, China

**Keywords:** subcutaneous fat, visceral fat, intra-pancreatic fat, MRI, Ethnicity

## Abstract

Anthropometric indices, such as body mass index (BMI), waist circumference (WC), and waist to height ratio (WHtR), have limitations in accurately predicting the pathophysiology of diabetes mellitus, cardiovascular diseases, and metabolic syndrome due to ethnic differences in fat distribution. Recent studies showed that the visceral adipose tissue (VAT) deposition and fat content of internal organs, most notably intra-hepatic and intra-pancreatic fat, has emerged as a more important parameter. In this study, we aimed to assess the coordination between the traditional anthropometric indices and the various fat depositions within different ethnicities in New Zealand. We recruited 104 participants with different ethnic backgrounds, including New Zealand Europeans, Māori (the indigenous people of New Zealand), Pacific Islanders (PI), and Asians. Their weight, height, and WC were measured, and subcutaneous, visceral, intra-hepatic, and intra-pancreatic fat depositions were obtained by magnetic resonance imaging (MRI). The result showed VAT, but not subcutaneous adipose tissue (SAT) depositions at all levels were significantly varied among the three groups. BMI was associated best with L23SAT in NZ Europeans (30%) and L45VAT in Māori/PI (24.3%). WC and WHtR were correlated well with L45SAT in the total population (18.8% and 12.2%, respectively). Intra-pancreatic fat deposition had a positive Pearson relationship with NZ European BMI and Māori/PI WC, but no regression correlation with anthropometric indices. Conventional anthropometric indices did not correspond to the same fat depositions across different ethnic groups.

## 1. Introduction

Distribution of fat is increasingly recognized as a key factor predisposing to type 2 diabetes, metabolic syndrome, and cardiovascular disease [1]. Fat around abdominal organs, termed “visceral adipose tissue” (VAT), is associated more closely with metabolic disorders than subcutaneous adipose tissue (SAT) [2,3]. VAT is regarded as more active than SAT in terms of triggering the release of mediators of inflammation and contributing to insulin resistance and deranged lipid metabolism [4,5,6,7]. Estimation of abdominal fat content is often done with the use of anthropometric indices, such as body mass index (BMI), waist circumference (WC), and waist-to-height ratio (WHtR). These indices are widely used as screening tools for adult cardiometabolic risk and are widely applied in the public as a way to predict metabolic syndrome and mortality [8]. However, in recent years, the fat content of internal organs, most notably intra-hepatic and intra-pancreatic fat, has emerged as a more important parameter in obesity, type 2 diabetes, and certain types of cancer [9,10,11,12,13]. 

Modern cross-sectional imaging, such as computed tomography (CT) and magnetic resonance imaging (MRI), are regarded as the gold standard as they can accurately quantify the specific distribution of adipose tissue in the abdomen and internal organs [14]. It is not considered practical to take a large number of images from each individual because it is costly and a time-consuming process, and thus it is crucial to establish a practical alternative method to estimate the fat content and distribution. The usefulness of different lumbar spine fat contents have reported weight differently in terms of evaluating the metabolic disorders, with some studies showing that the best single slice to evaluate VAT is at the two lowest vertebrae of the lumbar spine, L4-L5 [15,16,17]. Other studies have suggested that L1-L2 or L2-L3 can be better predictors of metabolic syndrome than L4-L5 [18,19,20]. This discrepancy can be explained, at least in part, by the fact that these associations vary between different ethnicities [21,22,23]. To date, there have been no published data on the best level to measure abdominal fat in Māori (the indigenous people of New Zealand) and Pacific Islander (PI) adults compared with other major ethnic groups in New Zealand (i.e., New Zealand (NZ) European and Asian). Further, there is a lack of data on ethnic variations in intra-pancreatic and intra-hepatic fat in New Zealand.

The aims of the study were to (1) compare SAT, VAT, and intra-hepatic and intra-pancreatic fat depositions in various ethnicities in New Zealand; (2) evaluate the ethnicity-specific associations between traditional anthropometric indices and MRI-derived fat content across the ethnicities; and (3) compare the usefulness of measuring fat depositions at different lumbar spine levels.

## 2. Materials and Methods

### 2.1. Study Design

This study was approved by the Health and Disability Committee and included healthy people aged 18 or above. Investigation of health and ethnic background was conducted to all patients before they went through MRI to make sure that they had no drug history and no malignancy, diabetes, coeliac disease, cystic fibrosis, chronic pancreatitis, pregnancy, or symptoms of upper abdominal pain and nausea. They also had no endocrine disorders, nor any history of acute infectious or inflammatory conditions requiring medical evaluation or treatment in the 3 months prior to the study date. All participants included in the study provided informed consent. Participants were excluded if they had general contraindications for MRI (such as metallic foreign body or electronic device implantation). Participants were grouped into NZ Europeans, Asians, and Māori/PI.

### 2.2. Anthropometric Measurements

BMI (kg/m^2^) and WC (cm) were measured at the time of MRI acquisition. WC was measured circling over light clothing from navel on participants. BMI was measured via a digital weight scale with a stadiometer (Health o meter Professional, 2013; Pelstar LLC, McCook, IL, USA).

### 2.3. MRI Acquisition

All participants underwent abdominal MRI at the Center of Advanced MRI (University of Auckland) with a field strength of 3.0 Tesla MAGNETOM Skyra scanner (Siemens, Erlangen, Germany). The axial T1-weighted volumetric interpolated breath-hold examination Dixon sequence was used in this study. The ratio of fat and water volume was used in analysis.

The HISTO (high-speed T2-corrected multi-echo single voxel spectroscopy) protocol in the Siemens Skyra 3.0 software was used to determine the intra-hepatic fat. It ran with 5 TEs ranging from 12 to 72 ms and a voxel of size 3 × 3 × 3 cm^3^, which was placed in the liver, avoiding major vessels and ducts. Proton density fat fraction (PDFF) was derived and corrected for fat and water transverse relaxation. The sequence was a 15 second breath-hold spectroscopy sequence for each patient. The HISTO automatic inline processing integrated the water and fat parts of the spectrum for individual echoes and performed a T2 relaxation correction. The fat signal was expressed as a percentage. A spectrum of the shortest TE and a list of quantification values for the individual echoes were added for quality control of the signal fitting.

**Figure 1:**
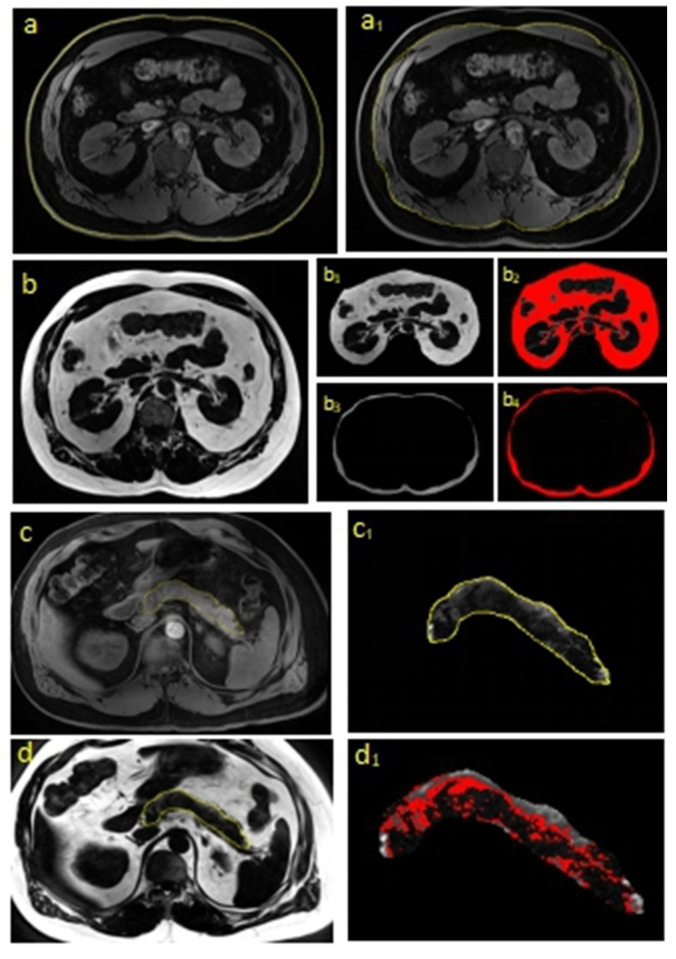
Water and fat content segmentation of subcutaneous adipose tissue (SAT), visceral adipose tissue (VAT), and intra-pancreatic fat using ImageJ software. (**a**,**a_1_**) The segmentation of subcutaneous and visceral water; (**b**–**b_4_**) the segmentation of subcutaneous and visceral fat; (**c**,**c_1_**) the segmentation of pancreatic water; (**d**–**d_1_**) the segmentation of intra-pancreatic fat.

To acquire data on SAT, VAT, and intra-pancreatic fat volume, we imported all MRI images into 3D-slicer software [24] to accurately locate and identify the first lumbar (L1) through to the fifth (L5) vertebral levels. Then, the corresponding water (the boundary size measurement) and fat scans at L12, L23, L34, L45, and the pancreas were separately cut out using ImageJ software (National Institutes of Health, Bethesda, MD, USA). The VAT compartment was then segmented and separated from SAT (Figure 1). The intra-pancreatic fat content was separated after comparing the corresponding water and fat scans. Water scan images were scaled by the free hand tool of ImageJ to visualize the boundary of each image for SAT, VAT, and the pancreas (Figure 1a,a_1_,c_1_), according to which the fat scan images were separated into equivalent sections (Figure 1b_1_,b_3_,d). The total sum of each slice boundary value was then multiplied by slice thickness to obtain total volume. Applying the threshold function in ImageJ on fat images, we converted the light gray images into binary images. The threshold was adjusted until the adipose tissue pixels were fully separated from the non-adipose tissue, such as blood vessels and the main pancreatic duct. The total pixel count of each slice image was measured (Figure 1b_2_,b_4_,d_1_) to yield the adipose tissue content of SAT, VAT, and pancreas. Total pixel counts in each slice were summed up, and then the ratio of fat to water was derived in each slice. The two series of images were analyzed and the ratio of the volume of fat content to water area was then calculated and defined as L12SAT, L23SAT, L34SAT, L45SAT, L12VAT, L23VAT, L34VAT, L45VAT, and PAT. The intra-hepatic fat content was read directly from the result of MR Spectrum, and termed as LAT.

### 2.4. Statistical Analysis

STATA for Windows (Stata Corp., 2019) and SPSS 23.0 for Windows (IBM Corp., 2015) were used for all statistical analyses. One-way ANOVA was applied to all variables to assess differences in baseline characteristics between the 3 ethnic groups. Data were presented as mean ± standard error. The independent *t*-test was then used to investigate the difference in each variable between each pair of the ethnic groups.

Firstly, to investigate the association between anthropometric indices and MRI-derived fat depositions in the entire cohort and each of the ethnic group, we used the Pearson’s correlation. Both *p-* and *r* values are presented to show the correlation between each variable.

Secondly, total and ethnicity-specific linear regression was performed in order to evaluate the usefulness of measuring fat contents at different lumbar spine levels, which represents the accurate regression relationship of the anthropometric measurements and each corresponding MRI scan. Age and sex variables were adjusted in analysis. Each adipose tissue volume percentage (L12SAT, L12VAT, L23SAT, L23VAT, L34SAT, L34VAT, L45SAT, L45VAT, LAT, and PAT) was treated as a dependent variable, and BMI, WC, and WHtR were used as independent variables in unadjusted and adjusted modes. Data are presented as β coefficients, 95% CI, and *R^2^* metric.

Thirdly, ethnicity-specific equations were derived from multicollinearity model in terms of estimating various lumbar spine VAT and PAT deposition from variables that do not require the use of MRI. The model included all predictor variables, and collinear variables (as shown by the highest variance inflation factor (VIF)) were removed one at a time until multicollinearity was minimized (VIF < 5). Back elimination method was applied to establish the equations that removed predict variables that did not significantly contribute to the model (*p* > 0.05).

## 3. Results

### 3.1. Baseline Characteristics

A total of 104 participants were recruited. In total, 53 (51%) were New Zealand Europeans, 30 (28.8%) were Māori/Pacific Islanders (Māori/PI), and 21 (20.2%) were Asians. Overall, there were 66 (63.5%) males and 39 (37.5%) females. The average age of all participants was 53.0 ± 1.6 years. Other characteristics are listed in Table 1.

### 3.2. Differences between the Ethnic Groups

Table 1 shows that there were significant differences between the ethnic groups in terms of both anthropometric measurements and all visceral adipose tissue percentage. However, there was no significant difference in terms of intra-hepatic and intra-pancreatic fat percentage. The *p*-values for different lumbar slices in evaluating visceral fat (i.e., L12VAT, L23VAT, L34VAT, L45VAT) were 0.019, 0.004, 0.014, and 0.039, respectively. Among them, the L23VAT was more significantly different (*p* < 0.01) than other levels. When different lumbar slices in evaluating subcutaneous fat were considered, only L12SAT was significantly different between the groups.

Further, the independent Student’s *t*-test was used to investigate the difference between each pair of groups (Table 2). There were differences when comparing NZ Europeans and Māori/PI, and Māori/PI and Asian groups in terms of all anthropometric indices, but there was no significant difference between NZ Europeans and Asians other than WC. The *p*-values for VAT and PAT reached the conventional level of statistical significance between NZ Europeans and Māori/PI, but the significant difference only existed between NZ Europeans and Asians in terms of LAT, and between Māori/PI and Asians in terms of L34VAT.

### 3.3. Correlations between Anthropometric Indices and MRI-Derived Fat Depositions across the Ethnicities

**Figure 2:**
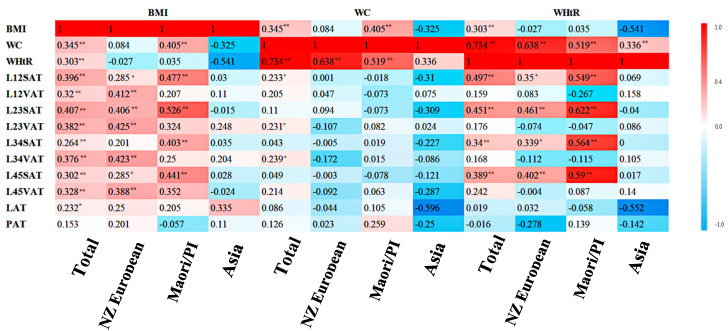
Pearson’s *r* values are presented, significant difference value is *p*; * and ** indicate significance at the 95% and 99% level, respectively.

The ethnicity-specific Pearson’s correlation coefficient (PCC) was calculated, and the r and the *p*-values are reported in the heat map (Figure 2). BMI, WC, and WHtR were used as independent variables, and each fat percentage was used as a dependent variable.

Overall, BMI was positively correlated with subcutaneous adipose tissues, visceral adipose tissues, and intra- hepatic fat, but not intra-pancreatic fat. The largest *r* value was 0.407 (for L23SAT). The WHtR was more significantly correlated (*p* < 0.01) with more dependent variables than WC. The WHtR was strongly correlated with each SAT, whereas WC was weakly correlated with VAT. The largest *r* value of WC was 0.239 (for L34VAT), and that of WHtR was 0.497 (for L12SAT).

In NZ Europeans, BMI was positively correlated with all the studied fat depositions. The largest *r* value was 0.425 (for L23VAT). The WHtR correlated with every SAT with the maximum *r* value of 0.461 (for L23SAT). WC had no significant correlation with any fat deposition.

In Māori/PI, BMI was more strongly correlated with SAT than VAT. The L23SAT correlated most with BMI (*r* value of 0.526). The WHtR in Māori/PI was significantly (*p* < 0.1) correlated with each single SAT. The L23SAT showed the largest *r* value of 0.622. WC did not correlate with either SAT or VAT, but correlated weakly with intra-pancreatic fat (*r* value of 0.259).

In Asians, there were weak but not significant correlations between traditional indices and fat depositions. BMI correlated with intra-hepatic fat (*r* value of 0.335) and L23VAT (*r* value of 0.248).

### 3.4. Usefulness of Measuring Fat Depositions at Different Levels

#### 3.4.1. Body Mass Index

In the overall cohort, BMI was associated with all slices of SAT and VAT, among which the L23SAT slice had the highest *R^2^*—up to 24%, with a β coefficient of 0.203 (*p* < 0.01), and the L45VAT slice had the highest *R^2^*—up to 13.9%, with a β coefficient of 0.237 (*p* < 0.01).

In NZ Europeans, BMI was associated with the L23SAT with the highest *R^2^*—up to 30%, with a β coefficient of 0.296 (*p* < 0.01); the L45VAT slice had the highest *R^2^*—up to 16.8%, with a β coefficient of 0.223 (*p* < 0.01). In Māori/PI, BMI was significantly associated with VAT only. The best level was L45VAT, with *R^2^* up to 24.3% and a β coefficient of 0.301 (*p* < 0.05). There were no significant associations between BMI and fat depositions in Asians (Table 3).

#### 3.4.2. Waist Circumference

In the overall cohort, WC had the strongest association with the L45SAT slice, with *R^2^* up to 12.2% and a β coefficient of 0.263 (*p* < 0.01). None of the associations were statistically significant in the three ethnic groups (Table 4).

#### 3.4.3. Waist to Height Ratio

In the overall cohort, WHtR had the strongest association with L12SAT (with *R^2^* of 23%, β coefficient of 0.196), L23SAT (with *R^2^* of 19.3%, β coefficient of 0.153), and L45SAT (with *R^2^* of 18.8%, β coefficient of 0.14). None of the associations were statistically significant in the three ethnic groups (Table 5).

### 3.5. Ethnicity-Specific Predictive Equations

Due to the weak correlation between PAT and LAT deposition and anthropometric indices, we only constructed ethnicity-specific equations to estimate the relationship between anthropometric indices and different level VAT depositions. In NZ Europeans, age, sex, and BMI were significantly correlated with each layer of VAT (Table 6). The derived equation in NZ Europeans was calibrated with each MRI-derived VAT deposition, and the *R^2^* values were 0.236, 0.263, 0.325, and 0.486 for L12VAT, L23VAT, L34VAT, and L45VAT, respectively. In Māori/PI, BMI and sex were both significant factors that were associated with all levels of VAT deposition, except L12VAT (Table 7). The calibrated *R^2^* values were 0.282, 0.336, and 0.383 for L23VAT, L34VAT, and L45VAT, respectively. In Asians, there was no significant association between VAT and anthropometric indices, which enabled a predictive equation.

## 4. Discussion

The study reported four key findings. Firstly, both SAT and VAT volume in Māori/PI was significantly higher than in the other two ethnic groups. Asians had a VAT fat volume similar to Māori/PI, especially in low abdominal slices, but had lower SAT fat volume. The Asian group had the highest PAT and LAT volumes. Secondly, the correlation results showed some significant ethnicity-specific relationships between anthropometric indices and fat depositions. Ethnicity-specific equations that predict MRI-derived VAT depositions from anthropometric indices were developed. Thirdly, the study showed no correlation between traditional anthropometric indices and intra-hepatic and intra-pancreatic fat depositions. Lastly, the power of the usefulness of fat deposition from different lumbar spine was varied between ethnic groups in terms of associating with anthropometric indices, which means ethnicity-specific evaluation should be fully weighted when diagnostic models are established. This MRI imaging study investigated the differences in distribution of adult adipose tissues between specific ethnicity groups—New Zealand Europeans, Māori/PI, and Asians. A state-of-the-art method was used in the present study that derives the fat and water volume from MRI images using threshold pixel contrast (which is different from previously published studies that used VAT and SAT areas but not their volume). Although using areas of VAT and SAT is timesaving, it lacks accuracy comparing with using volumes. In this study, the correlations between anthropometric indices (BMI, WC, and WHtR) and SAT, VAT from all intervals of lumbar discs (L12SAT, L23AT, L34AT, L45AT, L12VAT, L23VAT, L34VAT, and L45VAT), and the intra-organ fat content from liver and pancreas (LAT and PAT, respectively) were studied. Results were both age- and sex-unadjusted and adjusted.

A notable finding of this study was that VAT volume in the Māori/PI and Asian groups were similar and were significantly higher than in the NZ European group (despite having nearly the same SAT volume). The intra-hepatic fat deposition was higher in the Asian group, and the intra-pancreatic fat deposition was higher in Māori/PI. There were no statistically significant differences in SAT volume between the ethnicities. It is well established that SAT’s function is absorbing excess circulating free fatty acid and glycerol and storing triglycerides in adipocytes [25]. Once SAT is full of its lipid storage, fat begins to accumulate in fat compartments (especially VAT) [26]. It is believed that people with relatively higher VAT volume have a higher risk of developing metabolic disorders [1,27]. Furthermore, intra-pancreatic fat deposition has a negative correlation with insulin secretion, and it promises to become a novel and more accurate biomarker for metabolic disorders [28,29,30]. Previous studies also showed that genetic mutation has an impact on the lipid storage and end with more accumulation of VAT [31,32], which may interact with the ethnicity-specific VAT volume. Other similar studies of African Americans and Asians [33,34] also demonstrated that the ethnic diversity is associated with specific genetic background, which has considerable impact on the pattern of fat distribution. The results of this study are consistent with the investigation by the New Zealand Ministry of Health, according to which 1 in 3 New Zealand adults were confirmed as obese (2019), and Māori and Pacific Islanders had the highest rate of obesity (66.5% PI and 48.2% Māori), followed by NZ Europeans (29.1%) and Asians (13.8%) (New Zealand Health Survey: Ministry of Health, 2019). We showed that Māori and PI have significantly higher VAT and PAT volume compared with the other two groups. This suggests that visceral and intra-pancreatic fat depositions are likely to be the key at depots that are highly correlated with the morbidity of metabolic diseases. Genetic studies are now warranted to investigate metabolism-related gene expression patterns in Māori and PI.

The present study also investigated the strength of correlation between each fat deposition and anthropometric indices in the three groups. In addition to the two traditional indexes (BMI and WC), we also evaluated waist to height ratio (WHtR) as the third index, as there are published studies showing that WHtR is a better anthropometrical index than BMI and WC [8,35]. The results of this study did not support this finding, and showed that, in the overall cohort, WHtR had the weakest association with each SAT level. BMI was correlated with both SAT and VAT (and better with SAT than VAT), but did not correlate with LAT and PAT. WC correlated with SAT only—this finding is in line with studies in African Americans and Filipinos [36,37]. The above findings suggest that the anthropometric indices can roughly evaluate the adipose tissue distribution in people (mainly SAT) but do not have enough accuracy in estimating visceral adiposity across all ethnic groups. It is worth mentioning that growing evidence shows that intra-pancreatic fat is strongly associated with obesity, metabolic syndrome, type 2 diabetes mellitus, pancreatic exocrine dysfunction, acute pancreatitis, and pancreatic cancer [38,39,40,41]. Unfortunately, our study showed that there was no correlation between any anthropometric index and PAT. This highlights the need to develop a more accurate method to estimate intra-pancreatic and visceral fat deposition, taking into account different ethnic backgrounds. Given that deep machine learning holds promise in biomedical research [42,43] (including medical image recognition [44]), future studies will likely focus on developing integral machine learning system to recognize the fat depositions and their links with metabolic disorders.

The limitations of this study were as follows. Firstly, age and sex—the crucial determinants of adipose distribution—were not evenly distributed between the groups due to the randomness of participant recruitment. However, they were adjusted statistically with specific models in this study, which are shown in Table 3, Table 4 and Table 5. Since the adjustment of age and sex was conducted in a small group of participants, the results observed between ethnic groups in this study may be related to different group characteristics. It may be worthwhile to conduct a study in the future wherein more participants of each ethnic group are recruited. Secondly, the size of groups was imbalanced. The participants from the NZ European group were significantly higher than other two groups, and thus the observation of the difference presented between ethnicity-specific groups may have been related to the sample size. However, the current study represented an analysis of consecutive unselected participants who met the study eligibility criteria. Hence, our study cohort was representative of the ethnic ratio of the general population in New Zealand. The presented analyses should be viewed as exploratory and hypothesis-generating. The conclusion of the study will be used to inform the design and power calculation of future purposely-designed studies on ethnic variations in abdominal fat depositions. Thirdly, diet and physical exercise—common factors that influence fat accumulation—were not considered. However, to the best of our knowledge, no published study has investigated these factors as confounders in ethnicity-specific analyses. Fourthly, this study did not study genetic variations between the groups [45]. Future studies should also focus on the correlation between genetic variations and patterns of fat deposition in different ethnic groups. Lastly, the present study did not directly establish a correlation between the relevant metabolism milieus and every fat deposition. This warrants investigations in the future.

In conclusion, VAT and PAT depositions in Māori/PI were found to be significantly higher than in NZ Europeans and Asians. Intra-hepatic fat volume in Asians was higher than in the other two groups, whereas VAT and intra-pancreatic fat depositions were comparatively higher than in NZ Europeans. BMI appeared to be the most useful anthropometric index in this study. All the studied anthropometric indices only reflected abdominal fat compartments in ethnic groups and had different correlations, but they did not represent intra-organ fat depositions. VAT and intra-pancreatic fat may be the most important fat compartments for predicting the risk of metabolic disorders in New Zealanders.

## Figures and Tables

**Table 1:** Characteristics of study participants.

Characteristics	Total	NZ European	Māori/Pl	Asian	*p*-Value ^1^
No. of participants	104	53	30	21	
Age (years)	53.0 ± 1.6	56 ± 2.4	57.7 ± 2.0	39.1 ± 2.6	0.0001 ***
Sex					0.092
Male	66	31	21	14	
Female	38	22	9	7	
Height (cm)	171.4 ± 1.1	172.4 ± 1.2	172.5 ± 2.2	167.2 ± 2.1	0.109
Weight (kg)	82.6 ± 1.9	80.7 ± 2.1	93.7 ± 3.6	68.4 ± 4.0	0.0002 ***
WC (cm)	100.0 ± 0.7	96.6 ± 0.6	109.0 ± 1.2	95.7 ± 0.5	0.0002 ***
BMI (kg/m^2^)	28.1 ± 0.6	27.1 ± 0.6	31.7 ± 1.3	25.4 ± 1.0	0.0001 ***
WHtR	58.6 ± 0.6	56.2 ± 0.5	63.6 ± 1.2	57.4 ± 0.7	0.0001 ***
L12SAT	22.2 ± 1.0	20.3 ± 1.3	26.3 ± 2.3	21.1 ± 1.8	0.035 **
L12VAT	31.7 ± 1.0	29.2 ± 1.2	35.4 ± 1.4	32.6 ± 1.8	0.019 ***
L23SAT	26.7 ± 1.1	25.1 ± 1.5	29.3 ± 2.4	27.0 ± 2.2	0.280
L23VAT	32.4 ± 1.0	29.2 ± 1.5	36.8 ± 1.7	34.0 ± 1.9	0.004 ***
L34SAT	32.7 ± 1.1	31.1 ± 1.5	34.5 ± 2.3	34.0 ± 2.5	0.360
L34VAT	34.0 ± 1.1	31.8 ± 1.6	39.3 ± 2.4	39.0 ± 2.4	0.014 ***
L45SAT	37.9 ± 1.2	36.6 ± 1.6	39.3 ± 2.4	39.1 ± 2.4	0.549
L45VAT	32.4 ± 1.0	30.4 ± 1.4	36.0 ± 1.6	32.6 ± 2.2	0.039 **
SATA	30.0 ± 1.1	28.5 ± 1.4	32.4 ± 2.2	30.3 ± 2.1	0.291
VATA	32.6 ± 0.9	30.2 ± 1.4	36.8 ± 1.4	32.9 ± 1.9	0.008 ***
LAT	9.9 ± 1.0	8.2 ± 1.0	11.0 ± 1.9	13.5 ± 3.5	0.125
PAT	8.2 ± 0.4	7.5 ± 0.5	9.4 ± 0.6	8.0 ± 0.9	0.062

^1^ One-way ANOVA significant difference value is *p*, * *p* < 0.1, ** *p* < 0.05, *** *p* < 0.01. Data are presented as mean ± standard error. WC, BMI, and WHtR represent waist circumference, body mass index, and weight to height ratio, respectively. L12-L45 represent vertebrae of the lumbar spine. SAT and VAT stand for the percentage of subcutaneous and visceral fat, respectively. SATA and VATA are the mean values of all SAT and VAT volume, respectively. LAT and PAT stand for intra-hepatic and intra-pancreatic fat percentage, respectively (the same applies in the subsequent tables).

**Table 2:** Results of Student’s *t*-test for equality of means between the groups.

Anthropometric Indices and Fat Deposition	NZ European vs. Māori/Pl	NZ European vs. Asian	Māori/Pl vs. Asian
BMI	3.086 ***	1.471	3.0478 ***
WC	10.639 ***	2.208 **	12.539 ***
WHtR	6.796 ***	0.785	5.096 ***
L12SAT	2.362 ***	0.353	1.757
L12VAT	2.874 ***	1.287	1.101
L23SAT	1.684	0.651	0.864
L23VAT	3.07 ***	1.767	1.028
L34SAT	0.199	1.043	0.278
L34VAT	1.478 ***	0.224	2.227 **
L45SAT	1.184	0.818	0.256
L45VAT	2.431 ***	0.860	1.148
LAT	1.565	1.454 *	0.541
PAT	2.256 **	0.546	1.243

Student’s *t*-test significant difference value is *p*; *, **, and *** indicate significance at the 90%, 95%, and 99% levels, respectively.

**Table 3:** Regression result for ethnicity-specific associations between BMI and fat depositions.

Ethnicity Group	L12SAT	L23SAT	L34SAT	L45SAT	L12VAT	L23VAT	L34VAT	L45VAT
**Unadjusted**								
**Total**	0.225 ***	0.209 ***	0.137 ***	0.147 ***	0.196 ***	0.218 ***	0.202 ***	0.200 ***
	(0.052)	(0.047)	(0.050)	(0.047)	(0.057)	(0.052)	(0.049)	(0.057)
**NZ European**	0.135 ***	0.164 ***	0.085	0.109 ***	0.176 ***	0.177 ***	0.163 ***	0.178 ***
	(0.064)	(0.052)	(0.058)	(0.051)	(0.055)	(0.053)	(0.049)	(0.059)
**Māori/PI**	0.283 ***	0.297 ***	0.240 ***	0.247 ***	0.185	0.215 *	0.203	0.282 *
	(0.098)	(0.090)	(0.102)	(0.095)	(0.174)	(0.144)	(0.153)	(0.153)
**Asian**	0.018	−0.007	0.015	0.012	0.062	0.135	0.091	−0.011
	(0.135)	(0.110)	(0.096)	(0.097)	(0.127)	(0.121)	(0.101)	(0.108)
**Adjusted for Age and Sex**								
**Total**	0.310 ***	0.203 ***	0.337 ***	0.229 ***	0.207 ***	0.237 ***	0.201 ***	0.237 ***
	(0.187)	(0.239)	(0.085)	(0.124)	(0.089)	(0.135)	(0.085)	(0.139)
**NZ European**	0.221 ***	0.296 ***	0.165 **	0.226 ***	0.190 ***	0.204 ***	0.204 ***	0.223 ***
	(0.108)	(0.300)	(0.056)	(0.165)	(0.126)	(0.146)	(0.115)	(0.169)
**Māori/PI**	0.209	0.318	0.077	0.131	0.315*	0.271 **	0.275 *	0.301 **
	(0.149)	(0.196)	(0.118)	(0.137)	(0.228)	(0.238)	(0.223)	(0.243)
**Asian**	0.073	0.038	0.062	0.063	0.064	0.174	0.105	0.004
	(−0.111)	(−0.119)	(−0.111)	(−0.112)	(−0.110)	(−0.031)	(−0.066)	(−0.123)

*, **, and *** indicate significance at the 90%, 95%, and 99% levels, respectively. *R^2^* are reported in parentheses.

**Table 4:** Regression result for ethnicity-specific associations between WC and fat depositions.

Ethnicity Group	L12SAT	L23SAT	L34SAT	L45SAT	L12VAT	L23VAT	L34VAT	L45VAT
**Unadjusted**								
**Total**	0.133 ***	0.040	0.033	0.029	0.120 *	0.111 *	0.149 *	0.0100
	(0.065)	(0.060)	(0.061)	(0.057)	(0.070)	(0.065)	(0.061)	(0.070)
**NZ European**	0.047	0.012	0.017	0.043	0.008	0.002	0.012	−0.065
	(0.062)	(0.053)	(0.055)	(0.049)	(0.056)	(0.054)	(0.050)	(0.059)
**Māori/PI**	−0.050	−0.088	−0.059	−0.077	−0.052	−0.076	0.015	0.025
	(0.079)	(0.074)	(0.079)	(0.074)	(0.126)	(0.107)	(0.112)	(0.115)
**Asian**	0.008	−0.004	0.012	0.024	−0.012	−0.028	−0.005	0.016
	(0.056)	(0.046)	(0.040)	(0.040)	(0.053)	(0.052)	(0.043)	(0.045)
**Adjusted for Age and Sex**								
**Total**	0.196 ***	0.153 ***	0.115 **	0.140 ***	0.083	0.073	0.096 *	0.082
	(0.230)	(0.193)	(0.164)	(0.188)	(0.147)	(0.135)	(0.158)	(0.146)
**NZ European**	0.073	0.055	0.026	0.077	0.046	0.029	0.059	−0.025
	(0.231)	(0.225)	(0.213)	(0.240)	(0.223)	(0.215)	(0.228)	(0.213)
**Māori/PI**	0.117	0.198	0.120	0.149	−0.118	−0.093	−0.061	0.029
	(0.244)	(0.288)	(0.255)	(0.290)	(0.253)	(0.250)	(0.230)	(0.221)
**Asian**	0.034	−0.06	0.016	0.020	0.066	0.017	0.041	0.068
	(0.300)	(0.297)	(−0.111)	(0.296)	(0.319)	(0.297)	(0.310)	(0.332)

*, **, and *** indicate significance at the 90%, 95%, and 99% levels, respectively. *R^2^* are reported in parentheses.

**Table 5:** Ethnicity-specific associations between WHtR and fat depositions.

Ethnicity Group	L12SAT	L23SAT	L34SAT	L45SAT	L12VAT	L23VAT	L34VAT	L45VAT
**Unadjusted**								
**Total**	0.235 ***	0.187 ***	0.159 ***	0.167 ***	0.069	0.063	0.078 *	0.098 *
	(0.044)	(0.041)	(0.043)	(0.040)	(0.053)	(0.049)	(0.046)	(0.052)
**NZ European**	0.0170 ***	0.148 ***	0.134 ***	0.158 ***	0.028	0.001	0.007	−0.015
	(0.055)	(0.047)	(0.050)	(0.043)	(0.054)	(0.052)	(0.048)	(0.058)
**Māori/PI**	0.200 ***	0.205 ***	0.187 ***	0.194 ***	−0.172	−0.092	−0.059	0.056
	(0.066)	(0.061)	(0.068)	(0.063)	(0.117)	(0.102)	(0.108)	(00.110)
**Asian**	0.087	0.027	0.035	0.035	0.042	0.009	0.040	0.094
	(0.102)	(0.084)	(0.074)	(0.074)	(0.098)	(0.096)	(0.079)	(0.081)
**Adjusted for Age and Sex**								
**Total**	0.150 **	0.160 **	0.149 **	0.263 ***	0.078	0.070	0.110	0.059
	(0.057)	(0.058)	(0.054)	(0.122)	(0.028)	(0.027)	(0.041)	(0.023)
**NZ European**	0.091	0.034	0.038	0.086	0.032	0.031	0.059	−0.054
	(−0.017)	(−0.041)	(−0.040)	(−0.011)	(−0.041)	(−0.041)	(−0.229)	(−0.033)
**Māori/PI**	0.120	−0.016	0.073	−0.001	−0.109	−0.091	−0.029	0.003
	(0.007)	(−0.018)	(−0.006)	(−0.018)	(0.010)	(0.010)	(−0.016)	(−0.018)
**Asian**	−0.024	−0.052	−0.009	0.017	−0.002	−0.023	0	0.017
	(−0.108)	(−0.076)	(−0.114)	(−0.111)	(−0.115)	(−0.106)	(−0.115)	(−0.108)

*, **, and *** indicates significance at the 90%, 95%, and 99% levels, respectively. *R^2^* are reported in parentheses.

**Table 6:** Ethnicity-specific equations to estimate VAT depositions for NZ Europeans.

Correlation Factors	VIF	Equation
Age, years	1.061 *	L12VAT = 0.405 (age) − 0.565 (sex) + 0.347 (BMI) L23VAT = 0.460 (age) − 0.603 (sex) + 0.373 (BMI) L34VAT = 0.571 (age) − 0.612 (sex) + 0.348 (BMI) L45VAT = 0.464 (age) − 0.558 (sex) + 0.346 (BMI)
Sex	2.987 *	
BMI, kg/m^2^	1.679 *	
WC, cm	1.953	
WHtR	3.306	

* is statistical significance (*p* < 0.05). In the equation, sex: men = 1, women = 0. BMI: body mass index; WC: waist circumference; WHtR: waist to height ratio; PI: Pacific Islanders; VAT: visceral adipose tissue; VIF: variance inflation factor of VAT.

**Table 7:** Ethnicity-specific equations to estimate VAT depositions for Māori/PI.

Correlation Factors	VIF	Equation
Age, years	1.057	L23VAT = 0.615 (BMI) − 0.34 (sex) L34VAT = 0.518 (BMI) − 0.27 (sex) L45VAT = 0.642 (BMI) − 0.31 (sex)
Sex	2.987 *	
BMI, kg/m^2^	1.679 *	
WC, cm	1.953	
WHtR	3.306	

* is statistical significance (*p* < 0.05). In the equation, sex: men = 1, women = 0. BMI: body mass index; WC: waist circumference; WHtR: waist to height ratio; PI: Pacific Islanders; VAT: visceral adipose tissue; VIF: variance inflation factor of VAT.
